# Post-diagnostic allied health interventions for people with dementia in Australia: a spotlight on current practice

**DOI:** 10.1186/s13104-019-4588-2

**Published:** 2019-09-04

**Authors:** Monica Cations, Gorjana Radisic, Lenore de la Perrelle, Kate E. Laver, The Agents of Change Collaborative Group, Karen Shepherd, Fiona Methorst, Emma Baldwin, Danelle Maher-Norris, Joyce Gibson, Ebony Marsh, Wendy Brown, Jaimee Palagyi, Pamela Maryse Arndt, Kerri-Anne Vladcoff, Mariana Perez Sabja, Emily Caruana, Jean Tung, Jessica Doljanin, Jennifer Anderson, Jennifer Brittain, Lucy Comerford, Allison Wharley, Erin McKenzie, Su-Min Wong, Megan Seeliger, Vilma Delgado, Cassandra Kaizik, Katherine Kuo, Anne Millen

**Affiliations:** 1 College of Medicine and Public Health, Flinders University, Bedford Park, SA Australia; 2 Cognitive Decline Partnership Centre, The University of Sydney, Camperdown, NSW Australia

**Keywords:** Dementia, Post-diagnosis care, Exercise, Occupational therapy, Carer support, Aged care, Allied health

## Abstract

**Objective:**

The Clinical Practice Guidelines for Dementia in Australia provide evidence-based recommendations for the assessment, diagnosis, and care of people with dementia and their informal carers. The extent to which current Australian post-diagnosis care reflects these recommendations is not well understood. This brief report provides a snapshot of current practice related to three key recommendations from the Guidelines: occupational therapy, exercise, and informal carer support.

**Results:**

Nursing (*n *= 3) and allied health clinicians (*n *= 29) provided data about 1114 consultations with people with dementia and/or informal carers over a 9-month study period. Results showed that delivery of evidence-based dementia care remains a significant challenge in Australia. Clinicians found it difficult to tailor exercise interventions to overcome cognitive and organisational barriers to adherence during and between consultations. Occupational therapists primarily focussed on functional assessment rather than on delivering evidence-based interventions. Clinicians also found it difficult to identify and address the array of needs reported by informal carers, especially when the person with dementia is present during the consultation. Though these results are reported by a selected sample, they emphasise the need for innovative knowledge translation strategies to facilitate widespread quality improvement in post-diagnosis dementia care.

*Trial registration* Registered with the Australian New Zealand Clinical Trials Registry 21 February 2018 (ACTRN12618000268246)

## Introduction

Post-diagnosis care for people with dementia in Australia has been criticised for being insufficiently available, fractured, and focussed on managing impairments rather than promoting wellbeing [1, 2]. Rates of dementia diagnosis remain low [3] and services available after diagnosis will depend on demographic and practitioner factors [4]. These problems are common to other high-income countries internationally [5] though are exacerbated by Australia’s very low population density [6]. Despite being recommended as first-line treatment, non-pharmacological interventions are under prescribed [7]. Policy makers and people with dementia are promoting a ‘reablement approach’ that prioritises timely diagnosis, multidisciplinary early intervention, and ongoing access to non-pharmacological interventions such as occupational therapy, exercise, and carer support [2, 8].

Consistent with this, the Clinical Practice Guidelines for Dementia in Australia (the Guidelines, [7]) were developed to provide evidence-based recommendations for the assessment, diagnosis, and care of people with dementia and their informal carers. Three key recommendations from the guidelines specified that:

1. People with dementia living in the community should be offered occupational therapy (reflecting evidence-based programs).
2. People with dementia should be strongly encouraged to exercise.
3. Carers and family of people with dementia should have access to programs that provide respite and support to optimise their ability to provide care for the person with dementia.

The extent to which current Australian post-diagnosis care reflects these recommendations is not well understood. Some evidence suggests that Australian occupational therapists working with people with dementia prioritise home safety assessment at the expense of intervention [9]. However, much remains unknown including the typical needs of occupational therapy clients with dementia, the nature of interventions trialled, and referrals made. While international research has identified that people with dementia are not routinely encouraged to exercise [10], Australian practice patterns have not been formally established. Similarly, research from the United Kingdom suggests that service providers find it difficult to assess and respond to informal carer needs because of a focus on the needs of the person with dementia [11]. The extent to which similar issues are encountered in Australia is not known. These gaps in knowledge reflect a need to better understand the nature of current practice in more depth than is available in routinely collected data. While efforts are underway to establish a national registry for dementia care in Australia, such registries rarely capture detailed information about allied health and nursing practice.

As part of a larger implementation research project, described in detail elsewhere [12], we collected data from a sample of allied health and nursing professionals about the content of their consultations with people with dementia and their carers. Pre-intervention data for this project provides insight into some of the barriers to quality care in this context and can guide research and quality improvement efforts to where they are needed most. As such, this brief report summarises the baseline data of an 18-month translational research project for which health professionals provide detailed descriptions of their practice with people with dementia and their informal carers each month.

## Main text

### Method

The aim of the translational research project from which these data are taken is to improve adherence to the three key recommendations from the Guidelines listed above. We have established three Quality Improvement Collaboratives (QICs) in which health professionals complete a structured online education package and develop a unique quality improvement plan for their work site. Clinicians then meet monthly with the other members of their collaborative for shared brainstorming to overcome any barriers to implementing their plan. Data presented here were conducted prior to clinicians participating in the online education package.

Each month, participating clinicians complete a checklist about their first 10 consecutive consultations with people with dementia (Exercise, Occupational Therapy collaboratives) or informal carers (Carer Support collaborative). Ten consecutive consultations were considered an adequate sample of the clinician’s practice each month. Clinicians in all collaboratives were asked to report the date of birth and gender of the client, the nature of the consultation (i.e. initial assessment, part of treatment program), a brief summary of the consultation, whether a written treatment plan was provided, and any other resources or referrals provided. Clinicians in the Exercise collaborative additionally reported the activities they recommended, barriers to completion identified, and any strategies used or suggested to overcome these. Clinicians in the Occupational Therapy collaborative reported any assessments completed in the session and treatment strategies suggested. Clinicians in the Carer Support collaborative reported whether the carers’ needs were specifically addressed in the consultation and if so, the nature of these and strategies suggested.

Checklists were collated and analysed for themes by one author and checked by a second author. Both qualitative (e.g. the consultation summary) and quantitative data were examined for information about assessments, resources, and referrals provided. This information was grouped into categories descriptively (quantitative data) or using a content analysis approach of distilling words into content-related themes (qualitative data, [13]). A quantitative summary of these categories is presented. Ethical approval for this study was granted by the Southern Adelaide Clinical Human Research Ethics Committee on 21 August 2017 (HREC/17/SAC/88).

### Results

Thirty-eight implementation clinicians joined the QICs and 32 provided data that is included here (Table 1). All but one clinician is female and 21.9% work in regional, rural, or remote areas. Clinicians were relatively evenly dispersed across acute, community, and residential settings. Most did not have supervisory responsibilities for other staff. Checklists were submitted for 1114 consultations over the 9-month pre-intervention period. Clients described in the consultation checklists were 80 years old on average and more often female than male.

**Table 1:** Clinician characteristics

	*N* (%) or ẋ (SD)		
	Exercise *n *= 14	Carer support *n *= 9	Occupational therapy *n *= 9
Female	14 (100%)	8 (88.9%)	9 (100%)
Regional/rural/remote	4 (28.6%)	1 (11.1%)	2 (22.2%)
Profession			
Physiotherapy	12 (85.7%)	0 (0%)	0 (0%)
Occupational therapy	0 (0%)	2 (22.2%)	9 (100%)
Nursing	1 (7.1%)	2 (22.2%)	0 (0%)
Medicine	1 (7.1%)	0 (0%)	0 (0%)
Dietetics	0 (0%)	1 (11.1%)	0 (0%)
Health services	0 (0%)	4 (44.4%)	0 (0%)
Organisation type			
Public	5 (35.7%)	5 (55.5%)	5 (55.5%)
Private	4 (28.6%)	0 (0%)	0 (0%)
Not for profit	5 (35.7%)	3 (33.3%)	2 (22.2%)
Sole provider	0 (0%)	1 (11.1%)	2 (22.2%)
Service setting			
Acute	1 (7.1%)	0 (0%)	1 (11.1%)
Sub-acute/transition care	2 (14.3%)	0 (0%)	1 (11.1%)
Community/outpatient	3 (21.4%)	8 (100%)	5 (55.6%)
Residential	8 (57.1%)	0 (0%)	0 (0%)
Residential and community	0 (0%)	0 (0%)	2 (22.2%)
Median number of clients with dementia/carers per week (range)^a^	15 (7–40)	20 (2–42)	10 (3–38)
Supervisory responsibilities	4 (28.6%)	4 (44.4%)	0 (0%)

^a^Estimated by clinicians at the start of the project

Features of the consultations are described in Table 2.

**Table 2:** Consultation features

	*N* (%) or ẋ (SD)		
	Exercise (*n *= 620)	Carer support (*n *= 250)	Occupational therapy (*n *= 244)
Client age (years)	85.6 (9.5)	68.9 (11.3)	84.6 (9.6)
Female	372 (60%)	151 (60%)	142 (58%)
Consultation conducted^a^			
With person with dementia only	471 (76%)	N/A	98 (40%)
With carer only	N/A	93 (44%)	N/A
With PwD and carer together	149 (24%)	120 (56%)	146 (60%)
Assessment completed^b^			
Home environment/equipment	0 (0%)	28 (11%)	99 (41%)
Functioning (including falls, mobility)	379 (61%)	8 (8%)	85 (35%)
Service needs/safety to remain at home	0 (0%)	72 (29%)	85 (35%)
Cognition (including diagnostic assessment)	0 (0%)	78 (31%)	61 (25%)
Driving	0 (0%)	0 (0%)	2 (1%)
Carer stress	0 (0%)	108 (43%)	10 (4%)
Resources provided^b^			
Written treatment/strategies/activity plan	333 (54%)	93 (37%)	65 (27%)
Equipment prescription	60 (10%)	0 (0%)	115 (47%)
Written psychoeducation	84 (14%)	38 (15%)	37 (15%)
Referral	11 (2%)	128 (51%)	12 (5%)
Carer education	192 (31%)	79 (32%)^c^	146 (60%)
Referrals provided^b^			
GP or medical specialist	2 (0.3%)	10 (4%)	0 (0%)
Carer support/behaviour management	2 (0.3%)	80 (32%)	2 (0.8%)
Respite	2 (0.3%)	23 (9%)	2 (0.8%)
Another allied health service	2 (0.3%)	6 (2%)	3 (1%)
Home care services	2 (0.3%)	33 (17%)	5 (2%)

^a^Carer support *n *= 19 not reported

^b^May not add to 100% as clinicians may endorse more or less than one response

^c^Includes education regarding problem solving and strategies to manage changed behaviour

#### Exercise

Consultations by clinicians in the Exercise collaborative commonly included one or more recommendations of strength training (55%), balance training (37%), or cardiovascular training (53%). Mobility assessment (61%) was the most common reason for referral and massage was commonly provided (12%). Clinicians identified several barriers to the person with dementia completing physical exercise including insufficient availability of physical assistance (22%), low motivation or behaviour change (22%), cognitive impairment (9%), and inadequate therapist time to explain the activity (3%). Behavioural strategies to overcome these barriers were implemented in 54% of cases. These most often included using a family or paid carer to encourage the person (45%), written or verbal reminders (14%), and modifying the timing or type of activity (10%).

#### Carer support

Carers seen by clinicians in the Carer Support collaborative were mostly spouses (54%) or children (35%) of people with dementia; the remainder were other relatives or friends. Clinicians were not able to discuss carer needs in 22% of consultations because of sensitivities associated with having this discussion in front of the person with dementia or because there was not enough time. In cases where carer needs could be discussed, topics included carer stress (45%), inadequate support services (26%), difficulty managing challenging behaviours (21%), and the need for better understanding of dementia (8%). Only 22 consultation summaries (8.8%) noted that the carer had no concerns and was managing well. Most consultations by clinicians in the Carer Support collaborative included resource provision, usually information about available services (69%), referral (51%), counselling (35%), problem solving strategies (24%), strategies to manage changed behaviour (20%), and advanced care planning (18%). Referral to respite was made in nine per cent of cases.

#### Occupational therapy

Consultations reported by clinicians in the Occupational Therapy collaborative were diverse and often included several components in a single consultation. Most common was one or more of assessment of needs (67%), equipment or assistive technology prescription (47%), falls or pressure injury prevention (31%), home modifications (32%), and advocacy (including family education and case management; 20%). Less common was dementia-specific intervention such as skill building, memory strategies, cognitive stimulation, and identifying and modifying meaningful activities (24% of consultations).

### Discussion

Improvements in post-diagnostic dementia care are a policy priority in Australia. The new Aged Care Quality Standards rolled out in July 2019 mandate that aged care providers demonstrate efforts to implement quality improvements into their services [14]. However, the benefits of these efforts are difficult to identify without a detailed understanding of current practices. The data presented here provide a unique snapshot. Though the data was collected by a highly selected sample, clinicians participating in the project are diverse in terms of their professional backgrounds, service settings, and organisation type. The services offered in their consultations were as expected given prior studies [9–11]. Their summaries suggest that delivering best-practice dementia care remains a significant challenge and that innovative knowledge translation strategies are needed to facilitate widespread quality improvement.

The results are consistent with international findings that clinicians find it difficult to overcome the barriers to physical activity for people with dementia [10]. A recent systematic review identified that cognitive impairment, lack of insight, carer stress, and behaviour change add to the barriers already experienced by older people without dementia [15]. Health professionals need to be creative and persistent to produce individually tailored interventions, and adherence is promoted by use of a learning or adaptation period, regular reminders via phone calls, pedometers, or exercise logs, and supervision [16]. Previous research has identified nihilism and a lack of confidence to implement such adaptations among physical therapists working with people with dementia [17]. Adaptations may also be unrealistic in the context of organisational and time constraints.

Results also support previous findings that the needs of an informal carer can be difficult to identify where they cannot have a private consultation with a professional [11]. Although experts recommend integrated care models in which the person with dementia and their informal carer are treated as a dyad [18], carer needs can be easily overshadowed by the relatively greater needs of the person they care for. In addition, a wide array of needs was reported by informal carers in this study, including service navigation, responding to behaviour change, problem solving, continence management, advance care planning, and managing their own health needs. Clinicians are required to be a ‘jack of all trades’ to ensure they can provide appropriate information and referral [19]. Referral to respite services was uncommon despite this being a key strategy for promoting carer wellbeing [7].

A 2011 survey of Australian occupational therapists identified that therapists spent most of their time with people with dementia conducting assessment and advising about environmental modification [9]. Results here demonstrate a similar pattern; dementia specific interventions were implemented in only 24 per cent of consultations. Evidence-based occupational therapy interventions for people with dementia and their informal carers are associated with improvements in wellbeing, confidence in managing behaviour change, and self-reported independence [20, 21]. Systematic efforts are needed to promote wider adoption of these interventions by occupational therapists.

Few effective strategies have been identified to bridge the ‘evidence-practice gap’ in dementia care. The translational research project from which this data was taken aims to assess the utility of QICs in this context. Implementation clinicians are upskilled in quality improvement theory and methods via a structured online education package and expert feedback. They provide mutual support while designing and implementing a bespoke quality improvement plan in their setting [12]. Changes in guideline adherence related to this implementation strategy will be published in due course.

## Limitations

There are limitations to this data. This is a selected sample of clinicians who are motivated to implement quality improvements in their practice. They are also self-reporting, so may overstate or overestimate the quality of their practice. However, that even highly performing clinicians experience barriers to best practice highlights the importance of quality improvement efforts in dementia care and the complexity of this type of work. Participating clinicians are also not wholly representative of the dementia care workforce in Australia. Broadly examining current practices with an unselected sample will be important to better understand the key gaps in practice. Finally, the data presented do not provide insight into the acceptability and uptake of support options provided to the service users. Service uptake is known to be low among people with dementia [22, 23] but is a key part of an effective evidence-to-practice pipeline [24]. Research regarding methods to improve uptake of non-pharmacological interventions in this population is recommended.

## Data Availability

Data presented here will be made publicly available on completion of the translational research study from which they are taken.

## Abbreviation

QICs: quality improvement collaboratives
